# Bile Duct Leaks from the Intrahepatic Biliary Tree: A Review of Its Etiology, Incidence, and Management

**DOI:** 10.1155/2012/752932

**Published:** 2012-05-08

**Authors:** Sorabh Kapoor, Samiran Nundy

**Affiliations:** Department of Surgical Gastroenterology and Liver Transplantation, Sir Ganga Ram Hospital, New Delhi 110060, India

## Abstract

Bile leaks from the intrahepatic biliary tree are an important cause of morbidity following hepatic surgery and trauma. Despite reduction in mortality for hepatic surgery in the last 2 decades, bile leaks rates have not changed significantly. In addition to posted operative bile leaks, leaks may occur following drainage of liver abscess and tumor ablation. Most bile leaks from the intrahepatic biliary tree are transient and managed conservatively by drainage alone or endoscopic biliary decompression. Selected cases may require reoperation and enteric drainage or liver resection for management.

## 1. Introduction

Bile leaks mainly result from injury to the extrahepatic bile duct during cholecystectomy [1–3]. A bile leak from the intrahepatic biliary tree is less frequent and generally follows liver surgery and after blunt or penetrating abdominal trauma [4–6]. Less commonly, bile leaks from the liver may result following drainage of a liver abscess or nonsurgical ablation of liver lesions. The majority of leaks are transient and resolve spontaneously or after nonsurgical interventions like endoscopic retrograde cholangiography and pancreatography (ERCP) with sphincterotomy and/or stenting [6–8]. A few will need operative correction. However, these intrahepatic bile duct leaks result in significant patient morbidity leading to a prolongation of hospital stay and increase in healthcare costs. Bile leaks following liver resection also increase mortality rates [7, 9]. In this paper we will discuss how bile leaks are defined paper classified, what their causes are, and how they should be managed.

## 2. Definition

The most common accepted definition of a bile leak requires the presence of the following:

1. bile discharge from an abdominal wound and/or drain, with a total bilirubin level of >5 mg/mL or three times the serum level,
2. intra-abdominal collections of bile confirmed by percutaneous aspiration,
3. cholangiographic evidence of dye leaking from the opacified bile ducts [10].

## 3. Classification

Nagano et al. have classified postoperative bile leaks into four types [10]:

*Type A*: minor leaks from small bile radicles on the surface of the liver which are usually self-limiting,

*Type B*: leaks from inadequate closure of the major bile duct branches on the liver's surface,

*Type C*: injury to the main duct commonly near the hilum,

*Type D*: leakage due to a transected duct disconnected from the main duct.

*Type A* leaks usually close spontaneously with external drainage although sometimes ERCP and sphincterotomy may be required.

*Types B and C* can be managed by ERCP and stenting combined with drainage of the bile collection.

*Type D* leaks require surgery and bilioenteric anastomosis or, if the draining segment is small, fibrin glue occlusion or acetic acid ablation. Sometimes operative excision of the excluded segment may be required [10, 11].

## 4. Postoperative Bile Leaks

### 4.1. After Surgery for Hydatid Cysts

The incidence of bile leaks after surgery for hydatid disease of the liver varies from 4% to 28% [12–16]. For superficial small cysts without any obvious cyst-biliary communication, the incidence is low but increases for deeply located cysts, right lobe cysts, and cysts with daughter and satellite cysts and a pre- or intraoperatively diagnosed cyst-biliary communications. Agarwal et al. reported a 16% incidence of bile leaks in their series of 86 patients that were operated on for hydatid cysts of the liver with the incidence of leaks being higher after conservative surgical procedures, such as removal of the endocyst alone, rather than after a pericystectomy [12]. During surgery for hydatid cysts, it is important to avoid coloured scolicidal agents which may make it difficult to identify bile leaks. There should be a meticulous search for leaks using white lap pads after cyst evacuation if conservative surgery is performed and transcystic saline injection/cholangiogram with ligation of any ducts with visible leaks. In cases with large cyst-biliary communications either T tube placement or cyst-biliary-enteric anastomosis should be considered [12–16].

 Even after intra operative testing and closure of any leaking ducts, small leaks are still seen in upto 5% cases. These are mostly self-limiting and can be managed with drainage alone, [17, 18]. In a series of 304 cases all 10 leaks detected spontaneously closed over 4–8 weeks with simple drainage [16].

**Figure 1:**
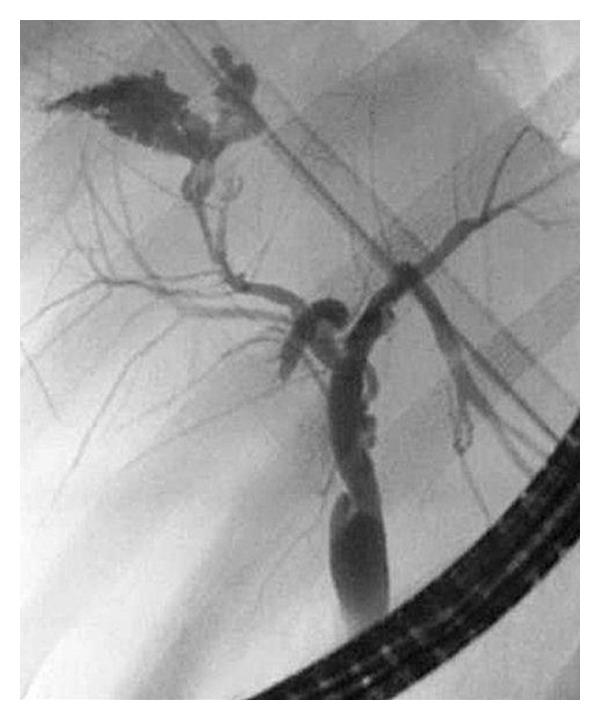
ERCP demonstrating bile leak in a patient who had cyst evacuation done for Hydatid cyst of the right lobe of liver.

 Although most leaks close spontaneously, they lead to a prolongation of hospital stay and may require additional procedures such as ultrasound guided drainage or ERCP with biliary decompression [12, 17, 18] (Figure 1). ERCP and sphincterotomy with or without stenting work by lowering the intrabile duct pressure and aid in early closure of leaks [17, 19–21]. Skroubis et al. have recommended indications for ERCP and sphincterotomy with/without stenting. They divided leaks into those with a low-output (<300 mL/day) and high-output (>300 mL/day) and recommended ERCP for high output fistulae persisting beyond 1 week or when low output fistulae continue to drain bile beyond 3 weeks [18]. Persistent fistulae despite ERCP and stenting or naso-biliary drainage require relaparotomy and enteric drainage [18, 19] (Table 1). 

### 4.2. After Liver Resections

The incidence of bile leaks following liver resection varies from 2% to 30% in different series [6, 10, 12]. The incidence depends on the type, extent and reason for liver resection.

#### 4.2.1. Liver Resection for Tumors

In the last two decades liver resections are being performed more frequently [22, 23] with a decreasing mortality—large volume centers reporting mortality rates of less than 5% [22–26]. However, the morbidity of the liver resection still remains in the range of 20–50% [27–29].

Bile leaks after liver resection in the absence of any biliary enteric anastomosis are a major cause of morbidity and lead to prolonged drainage, intra-abdominal collections, and abscesses. In addition, bile leaks also lead to prolongation of the hospital stay [6–9]. The reported incidence of bile leaks in various large series of hepatic resections varies from 2.5 to 12% [6, 7, 25, 30].

A bile leak rate of 8% was reported in a large series of 340 liver resections performed for hepatic malignancies [8]. In a retrospective analysis of 205 liver resections, Erdogan et al. reported bile leaks from the intrahepatic biliary tree in 7.5% patients—with a higher incidence of 9% after resections for malignant tumors compared to 4% leaks for benign lesions [31]—whilst Clarke et al. reported bile leak rates of 6% after elective liver resection also for benign tumours [32]. However, in another large study comprising 610 cases, no difference in bile leak rates was found for benign tumours compared to those done for malignancy [6]. Only resections for intrahepatic cholangiocarcinomas were found to be associated with higher bile leak rates [6, 8]. This may be related to the deep location of the tumor requiring a major hepatectomy and a dissection close to the major ducts and hilar plate [6–9]. Many authors have reported a lower incidence of postoperative bile leaks from the intrahepatic bile ducts when enucleation was performed for liver hemangiomas compared to resection [33–36]. However others have not found significant difference in leak rates for nonanatomical versus anatomical resections [6].

The *extent* and *type* of resection have also been reported to be related to the incidence of leaks. The incidence is higher after a central hepatectomy involving segments 4, 5, and 8, right anterior sectionectomy (segments 5 and 8), left trisectionectomy, isolated segment 4 resection, and caudate lobe resections [6, 7, 9, 10]. In addition, left hepatectomy is associated with higher bile leaks probably due to drainage from an aberrant right posterior duct joining the left duct [9] (Table 2).

Leak rates are also higher when hepatectomy is combined with bile duct resections and bilioenteric anastomoses [9]. Incidence of bile leaks is not significantly affected by the technique of pedicle division (whether extrahepatic or intrahepatic). Smyrniotis et al. found a similar incidence of bile leaks on a retrospective comparison of 100 hepatectomies performed with intrahepatic pedicle division compared to 50 hepatic resections done with extrahepatic pedicle ligation. However, bile leaks following intrahepatic ligation were mostly self-limiting and transient compared to leaks following extrahepatic pedicle ligation which were likely to be prolonged and often required ERCP and biliary decompression [42].

The *method of transection* does not seem to affect the leak rates. Thus there was no difference between using clamp crushing, the ultrasonic dissector, harmonic scalpel, tissue link dissecting sealer, or Ligasure for liver resection [43–45]. The incidence of bile leak is reported to be higher after Radio-Frequency- (RF-) assisted liver resection in some series [46, 47] but no difference was found in a Cochrane database systematic review comparing different parenchymal transection techniques [48].

The use of a stapler for parenchymal transection has also not been shown to result in an increase in the incidence of bile leaks. In a series of 62 consecutive liver resections using staplers, the incidence of bile leak was 3% [49]. Subsequently 2 large studies of 101 liver resections from Pittsburgh, USA, and 300 parenchymal transections from Heidelberg, Germany, using staplers reported leak rates of 1% and 8%, respectively [50, 51].

The initial fears regarding a higher rate of biliary leaks after *laparoscopic hepatectomy* have been unfounded. A review of 2804 cases of laparoscopic or laparoscopy-assisted liver resections published in 2009 reported bile leak rates of 1.5% [52]. The lower bile leak rates may be partially due to selection of less complex cases for laparoscopic liver resection and because these resections are mostly performed by experienced hepatobiliary surgeons with advanced laparoscopic training.

#### 4.2.2. After Donor Hepatectomy

Living donor hepatectomy is a special situation because surgery is performed in healthy individuals; hence, extra efforts are made to decrease postoperative morbidity that may affect recovery or a prolonged hospital stay. The reported incidence of bile leaks after donor hepatectomy varies from 0% to 9% [30, 37–41] (Table 3). The most common site of a bile leak is the cut surface of the liver, from small branches in the caudate lobe or from the hilar plate. The leaks are invariably self-limiting and respond to drainage alone. Posttransection assessment of the bile duct in the donor and sites of leak should be carefully done using a transcystic cholangiogram using radio-opaque contrast or by instillation of colored dyes such as methylene blue or indigo carmine [37].

## 5. Prevention of Bile Leaks after Liver Surgery

It is important to meticulously identify any leaking ducts during and after transection and carefully ligate them. Cholangiography is recommended for living donors to ensure that the main duct is not injured as well as to identify leaks [37]. Careful inspection of the residual cavity is also essential after conservative operations for hydatid cysts and simple cysts and after enucleation of hemangiomas [12, 13, 15, 53].

Posttransection testing of potential leaks by injecting saline, methylene blue, indigocarmine, or ICG (Indocyanine green) is recommended by many surgeons to identify any bile leaks from the cut surface or hilar plate which, if found, should be sutured. Most surgeons use transcystic duct saline injection which is able to identify significant leaks. [11]. The only randomised trial that assessed the efficacy of bile leak testing using saline found no benefit, however, the leaks rates were lower in both tested and non tested groups and both groups, had fibrin glue applied to the transection surface [57]. Subsequently a large nonrandomized series by Yamashita et al. reported no bile leaks in 102 consecutive liver resections after they started using intraoperative testing with transcystic saline injection compared to leak rates of 4.5% in 679 hepatectomies with no leak testing [7]. A new technique described by Japanese surgeons involves injection of indocyanine green (ICG) dye through the transcystic tube followed by fluorescent imaging. The authors reported that small leaks not identified by a leak test using saline could be detected using this technique [58]. ICG fluorescence cholangiography after hepatic resections in 52 cases was compared to a conventional leak test using ICG dye alone in 50 cases in another study. In the fluorescence group, additional leaks were seen in 25 patients that were subsequently ligated. Postoperative leaks occurred in 10% in a conventional leak test group compared to no leaks in the ICG fluorescence group [59]. However, ICG dye is not easily available everywhere and the technique also requires special fluorescence imaging equipment to be available in the operating room. In addition, the clinical impact of small blushes seen on fluorescence is not clear. Similar NIR (Near infrared imaging) has also been applied for intraoperative identification of the bile ducts, but the technique is still predominantly experimental and not available widely [60].

The use of fibrin glue or sealants may be beneficial in decreasing bile leaks as reported by a number of authors [6, 8, 61, 62]. Only one randomized trial looked at fibrin glue application on the cut surface and found lower drain bilirubin concentrations in the early postoperative period [63]. However, the benefit of fibrin glue and other topical sealant application to the resection margin in preventing bile leaks remains to be substantiated by properly designed trials [64].

## 6. Management of Disconnected Bile Ducts after Liver Resection

Bile leaks from disconnected ducts or excluded segment ducts after liver resection tend to be associated with persistent drainage or recurrent intra-abdominal collections. The usual cause is aberrant anatomy of the bile duct or a non anatomical resection resulting in a Type D Nagano fistula with disconnection of the biliary drainage of a portion of the remnant liver from the main substance while the vascularity of the parenchyma is maintained. ERCP in these cases does not demonstrate any leak in the presence of ongoing fistula output. Only a fistulogram or direct percutaneous cholangiogram of the involved segmental duct will demonstrate the excluded segment duct which does not have a connection with the main biliary tree. These cases usually require bilioenteric drainage or resection of the residual liver which is often difficult due to the presence of adhesions and sepsis [10, 54, 65].

**Figure 2:**
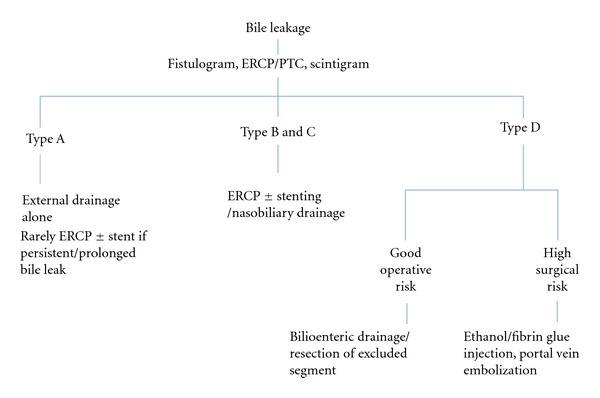
Classification based management of intrahepatic bile leaks.

A minimally invasive approach using fibrin glue, ethanol ablation of the draining liver segment, or portal vein embolization to induce atrophy of concerned liver segment has been proposed as less invasive alternatives to surgery (Table 4) (Figure 2) [7, 54–56].

## 7. Bile Leaks following Nonsurgical Procedures

**Figure 3:**
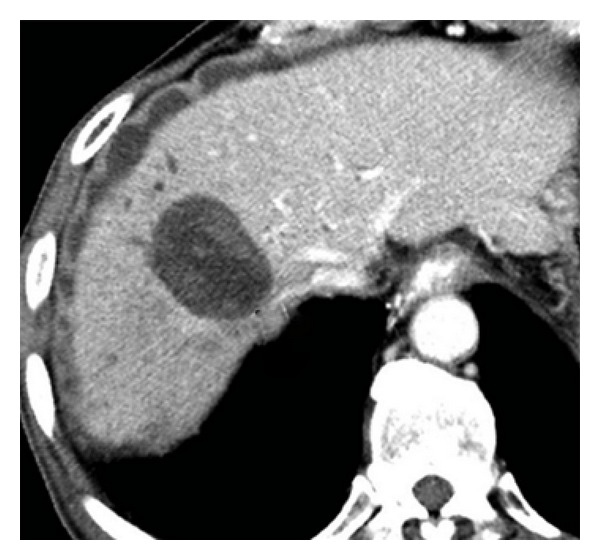
CT scan showing a biloma surrounding the ablated tumor after RF ablation.

*Radio frequency ablation (RFA)* is a commonly used technique for ablation of liver tumors, both metastatic and primary. Bile duct injury although common after this procedure usually does not manifest with leaks; most cases present with mild ductal dilatation on imaging (Figure 3). Major injury presenting with biloma or leaks is seen less often in 0.5% to 5% patients and occurs when the ablated area is centrally located near a major biliary radicle or when surface lesions are ablated. The differences in the reported incidence may also be related to the difference in the number and size of lesions ablated in different reports [66–69].

**Figure 4:**
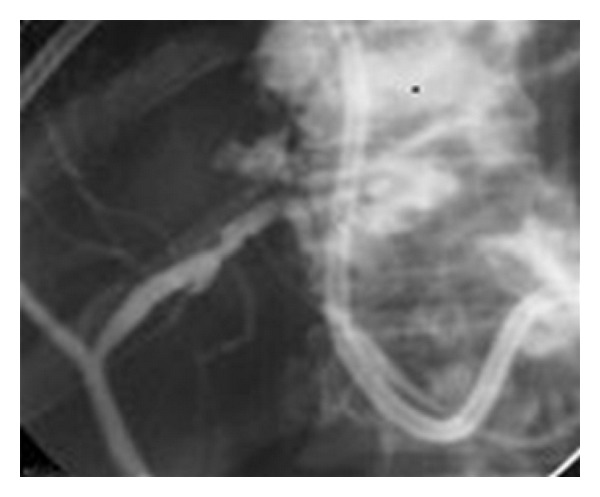
ERCP demonstrating bile leak into the abscess cavity after percutaneous liver abscess drainage.

Bile leak following *percutaneous drainage of a liver abscess* is often encountered. Some cases of intrahepatic biliary tree injury due to destruction of liver tissue including bile radicles and vascular channels by the inflammatory process may present with leakage of bile into venous channels and present as bilhemia (elevated bilirubin without a concomitant rise in serum transaminase levels). Bile leaks may complicate both amoebic and pyogenic liver abscesses with a reported incidence varying from 5 to 27% [70, 71] (Figure 4). In the majority of cases, the drainage gradually decreases and stops spontaneously; recalcitrant cases require ERCP and stenting or nasobiliary drainage for resolution. In a large series of 525 patients with liver abscesses managed at a single center, 26 patients with biliary fistula or bilhemia required ERCP and stenting/nasobiliary drainage with resolution occurring in all [72]. Recently the same group presented their updated results over 10 years with 38 out of 586 liver abscess patients requiring endoscopic stenting or nasobiliary drainage for bile leaks or jaundice (bilhemia) [73].

## 8. Intrahepatic Bile Duct Leaks after Noniatrogenic Trauma

**Figure 5:**
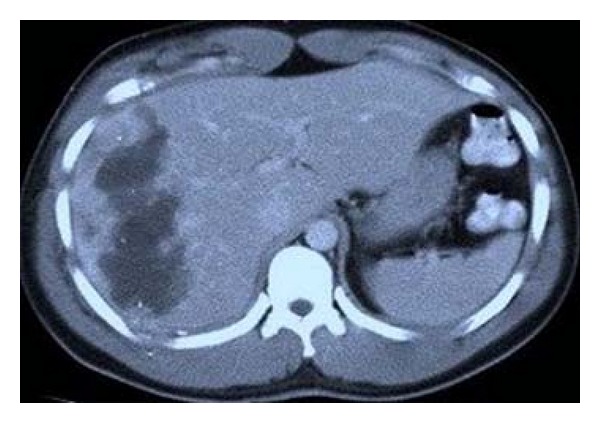
CT scan showing large hematoma in Right lobe of liver following blunt trauma. The hematoma was complicated by bilhemia.

Bile leaks can lead to significant morbidity after liver trauma. Following trauma and liver hematoma associated with injury to the intrahepatic bile ducts, the influx of bile into the hematoma may increase the pressure within it, leading to necrosis of the surrounding liver tissue and formation of a biloma [74] (Figure 5).

**Figure 6:**
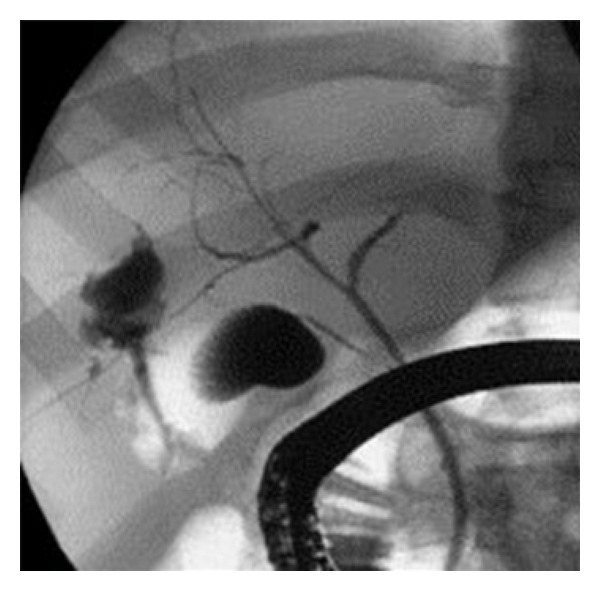
ERCP demonstrating bile leak following liver laceration. The patient was managed conservatively with endoscopic stent placement.

Leaks are more common after penetrating injuries, especially where damage control surgery and perihepatic packing has been done. Overall the incidence of intrahepatic bile duct injury after blunt trauma for all grades of injury varies from 2.8% to 7.4% [75, 76]. Most cases of bile duct injury after blunt trauma present as bilomas which can be managed conservatively. The pain associated with enlargement on imaging or the presence of infection is managed by percutaneous drainage in combination with ERCP [77, 78] (Figure 6). Leaks may also complicate high-grade liver injuries following blunt trauma with almost two-thirds of the patients who require surgery developing bile leaks compared to 17% in cases of high-grade liver injury where nonoperative management is successful [79].

Bile peritonitis which requires laparotomy and drainage may also be managed by a minimal invasive combination of laparoscopic lavage and ERCP decompression [80, 81]. Bile duct leaks may be delayed after blunt trauma as hematoma or liver lacerations may devitalize the parenchyma leading to biloma and leak/bilhemia. Follow-up imaging is therefore recommended beyond grade 2-3 liver trauma to evaluate the development of biloma, fluid collections, or vascular complications [82].

## 9. Conclusion

Extrahepatic bile duct injuries being more frequent often overshadow injuries to the intrahepatic bile ducts. The latter are, however, a significant cause of morbidity after liver surgery, blunt or penetrating trauma, and some nonsurgical ablative or drainage procedures. Determination of type of injury based on Nagano classification is useful in deciding the optimal management and the likelihood of success with conservative measures. While most intrahepatic bile duct leaks (Nagano Type A) are self-limiting and respond to external drainage, some major leaks (Nagano Types B and C) often require ERCP and stent placement in the common bile duct and a select few patients with Nagano Type D injury require surgical management in the form of bilioenteric anastomoses or liver resection.

## Figures and Tables

**Table 1:** Bile leaks following surgery for Hydatid cysts of liver.

Number	Author	*N* =	Bile leaks	Presentation	Management	Comments
(1)	Agarwal et al. [12]	86	14 (16%)	Bile cutaneous fistula 11; biloma 3	Spontaneous closure in 11; ERCP in 3	All leaks in conservative surgery group
(2)	Puliga et al. [13]	232	27 (11.6%)	—	—	25.2% leaks in conservative; 2.8% in radical
(3)	Unalp et al. [14]	183	24 (13.1%)	17 low output; 7 high output	17 spontaneous closure; 7 ERCP	All conservative surgery
(4)	Silva et al. [15]	30	7 (23.3%)	Bilio cutaneous fistula 7	Drainage alone	29 conservative; 1 radical
(5)	Skroubis et al. [18]	187	18 (10%)	3 bilomas; 1 bile peritonitis, 14 biliary fistulas (1 bronchobiliary)	13 drainage alone; 5 ERCP (including broncho biliary)	All conservative surgery

**Table 2:** Bile leaks after liver resection for benign and malignant tumors.

Number	Author	*N* =	Diagnosis	Bile leak	Comments
(1)	Capussotti et al. [6]	610	Benign disease 53; Malignant 557	22 (3.6%)	Fibrin glue protective; more leaks for peripheral hepatic cholangiocarcinoma and resections involving segment 4

(2)	Yamashita et al. [7]	781	Benign 69; malignant 712	31 (4%) Benign 2.9%; malignant 4.1%	Major hepatectomy including segment 4 and caudate higher risk; intraop leak test beneficial

(3)	Tanaka et al. [8]	363	26 (7.2%)	All malignant	Higher leaks for intrahepatic cholangiocarcinoma

(4)	Lo et al. [9]	347	Benign 62; malignant 285	28 (8.1%)	Higher leaks for left hepatectomy, left trisegmentectomy, older patients, and cholangiocarcinoma

(5)	Jarnagin et al. [25]	1803	Benign 161; malignant 1642	47 (2.6%)	Higher morbidity for complex resections and patient comorbidity

(6)	Imamura et al. [30]	825	Benign 31; malignant 794	77 (9.3%)	Higher leak for complex resections

(6)	Erdogan et al. [31]	205	Benign 70; malignant 135	13 (6.3%); benign 4.3%, malignant 7.4%	Presence of comorbidity and complex resections associated with higher morbidity

(7)	Clarke et al. [32]	49	All benign	3 (6.1%)	Low incidence of leaks for benign lesions

**Table 3:** Bile leaks after living donor hepatectomy.

Number	Author	*N* =	Bile leak	Comments
(1)	Imamura et al. [30]	174	11 (6.3%)	Right lobe donors higher leak rates
(2)	Chan et al. [37]	200 (all right lobes)	0	Meticulous ligation of all bile leaks during transaction
(3)	Cipe et al. [38]	140 (108 Right lobe)	13 (9.2%)	More leaks after right lobe
(4)	Iida et al. [39]	1262 (500 right lobes)	123 (9.7%); Right lobe 12.2%, left 4.9%	Right lobe higher biliary leaks
(5)	LaPointe Rudow et al. [40]	70 (all right lobes)	3 (4.3%)	None
(6)	Ghobrial et al. [41]	393	36 (9%)	None

**Table 4:** Management of Excluded segment (Nagano Type D) Bile leaks.

Number	Author	Diagnosis	*N* =	Number of resections/surgery	Management
(1)	Lo et al. [9]	Benign and malignant	2	347	Surgery
(2)	Nagano et al. [10]	Malignant	1	313	Surgery
(3)	Honoré et al. [54]	Malignant and benign	3	2409	Surgery
(4)	Yamashita et al. [7]		6	781	1 spontaneous closure with prolonged drainage and atrophy 3 Ethanol injection 1 balloon catheter occlusion
(5)	Tanaka et al. [8]	Malignant	2	363	Ethanol injection
(6)	Skroubis et al. [18]	Benign (all hydatid cysts)	1	187	Surgery
(7)	Kyokane et al. [55]	Malignant	1	—	Portal vein embolisation
(8)	Yamakado et al. [56]	Malignant	1	—	Portal vein embolisation
